# A Fluid-Driven Loop-Type Modular Soft Robot with Integrated Locomotion and Manipulation Capability

**DOI:** 10.3390/biomimetics8050390

**Published:** 2023-08-26

**Authors:** Xin Sui, Mingzhu Lai, Jian Qi, Zhiyuan Yang, Ning Zhao, Jie Zhao, Hegao Cai, Yanhe Zhu

**Affiliations:** 1 State Key Laboratory of Robotics and System, Harbin Institute of Technology, Harbin 150001, China; suixinhit@163.com (X.S.); 20b908059@stu.hit.edu.cn (J.Q.); yzy19962@live.com (Z.Y.); zhaoning1995@hit.edu.cn (N.Z.); jzhao@hit.edu.cn (J.Z.); hgcai@hope.hit.edu.cn (H.C.); 2 School of Mathematics and Statistics, Hainan Normal University, Haikou 571158, China; laimingzhu@126.com

**Keywords:** soft robot, modular robot, rhythm regulate, fluid element actuator, vision feedback

## Abstract

In nature, some animals, such as snakes and octopuses, use their limited body structure to conduct various complicated tasks not only for locomotion but also for hunting. Their body segments seem to possess the intelligence to adapt to environments and tasks. Inspired by nature, a modular soft robot with integrated locomotion and manipulation abilities is presented in this paper. A soft modular robot is assembled using several homogeneous cubic pneumatic soft actuator units made of silicone rubber. Both a mathematical model and backpropagation neural network are established to describe the nonlinear deformation of the soft actuator unit. The locomotion process of the chain-type soft robot is analyzed to provide a general rhythmic control principle for modular soft robots. A vision sensor is adopted to control the locomotion and manipulation processes of the modular soft robot in a closed loop. The experimental results indicate that the modular soft robot put forward in this paper has both locomotion and manipulation abilities.

## 1. Introduction

Soft robots have become a popular topic of research in the recent decades since they can imitate the behaviors of creatures with soft bodies in nature to tackle tasks that are difficult for those with rigid ones. There are two basic classifications for common soft robots: the manipulation classification and the locomotion classification. Soft robots in the category of locomotion mostly comprise of the function of crawling [1,2,3], running [4], jumping [5,6,7], rolling [8,9], and wriggling [10,11]; while soft robots in the category of manipulation primarily include adsorption [12,13], grasping [14,15], twining [16], twisting [17,18], and manipulation functions [19,20,21]. Soft actuators play a key role in determining the functionalities and motion modes of soft robots. They are different from rigid actuators as most soft actuators are driven by a fluid [22,23,24], and the actuation functions of soft actuators are strongly associated with initial structures. For this reason, the structure of a soft actuator should be determined at the design stage, representing a high-level requirement for designers that is inconvenient to modify afterwards. Hence, this work adopts a modularization method to design a soft actuator. The concept of modularization mainly entails the qualities of being reconfigurable, replaceable and reusable; that is, a soft actuator with a complex structure and inner cavities can be divided into a number of homogeneous actuator units, and the actuator units can be assembled in accordance with task scenarios to realize different actuating functions. This train of thought simplifies the design process and reduces the cost of maintenance and alteration.

Modular soft robots [25] are also designed based on analogous organisms found in nature, such as various annelids, snakes, worms, octopuses, and so on. Such kinds of animals actuate the segments of their bodies in particular rhythms; thus, their whole bodies locomote forward just like waves. Numerous researchers present works that consider biomimetic ideas. Rus [26] and her co-workers designed a pneumatic soft module that can be assembled into various configurations with the robots having corresponding locomotion patterns. Luo et al. [27,28] took a snake-like robot assembled using soft modules as their research object and conducted a profound study on the locomotion principle of that modular soft robot. Fei et al. [29,30,31] researched a spherical soft modular robot and modeled a nonlinear dynamic model of the multi-spherical module system so that the system was under steering control and avoided obstacles. Zou et al. [32] proposed an extendible soft actuator unit with units that can be connected using miniature magnets that are embedded in them. The entire soft robot system has the ability of omnidirectional locomotion. Jun Ogawa et al. [33] focused on the inner cavity structure of soft modules. Different inner cavities determine the different functions of soft modules, and soft modules can be assembled to conduct various tasks. However, there are few studies on soft robots with both locomotion and manipulation abilities [34]. In fact, the locomotion and manipulation functions are not strictly distinguished between the animals put forward above. Consider a snake, which uses its body to wrap its prey as well as wind itself ahead. The body’s segments perform dual roles as “hands” and “legs”, switching their roles according to the environment and task.

Consequently, a mobile soft manipulation platform (MSMP) with integrated locomotion and manipulation abilities using biomimetic principles and modularization is put forward in this paper. The MSMP is assembled via homogeneous pneumatic soft actuator units. To explicate our idea, our paper is organized according to the following sections and contains (1) a description of the design of the soft actuator unit and the MSMP; (2) the locomotion and deformation analyses of the MSMP; (3) a discussion of the control strategy of the MSMP; (4) the experimental evaluation and discussion; and (5) the conclusion.

## 2. Design of the Soft Actuator Unit and the MSMP

**Figure 1:**
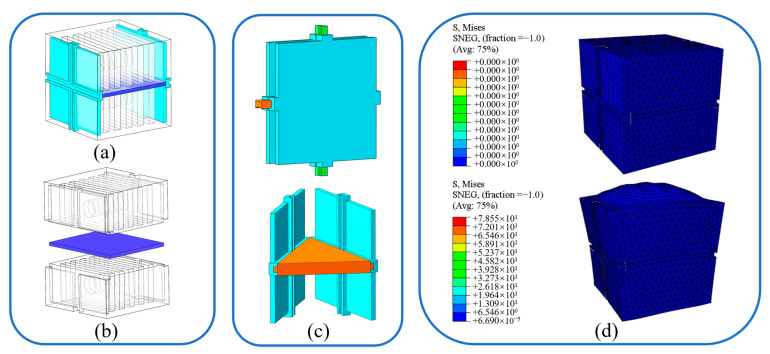
3D model of (**a**) the soft actuator unit; (**b**) the soft parts of the actuator unit; (**c**) the rigid connection mechanism; and (**d**) the deformation results of the soft actuator unit in ABAQUS.

The soft actuator unit described in this paper adopts the design principle of a fluid elastic actuator (FEA) and draws on the experience of the bi-directional deformation FEA presented by Rus [26]. Additionally, the bi-directional FEA is modified to have a cubic structure with a uniform side length so that the soft actuator unit can be extended in three-dimensional directions, as shown in Figure 1a. The soft part of the actuator unit is made of Ecoflex-0030 silicone rubber; is constructed via demolding; and the middle layer is made of polydimethylsiloxane (PDMS), as shown in Figure 1b. Plastic plates with special grooves are embedded in both sides of the soft part. When two actuator units are close enough to one another two grooves can be joined into a single specially formed hole which allows a connector with the same section shape to connect the two units together through insertion, as shown in Figure 1c. Figure 1d illustrates the result of the finite element analysis of the soft actuator unit in ABAQUS, version 2021. The upper chamber of the soft actuator unit is inflated at an air pressure level of 15 kPa.

**Figure 2:**
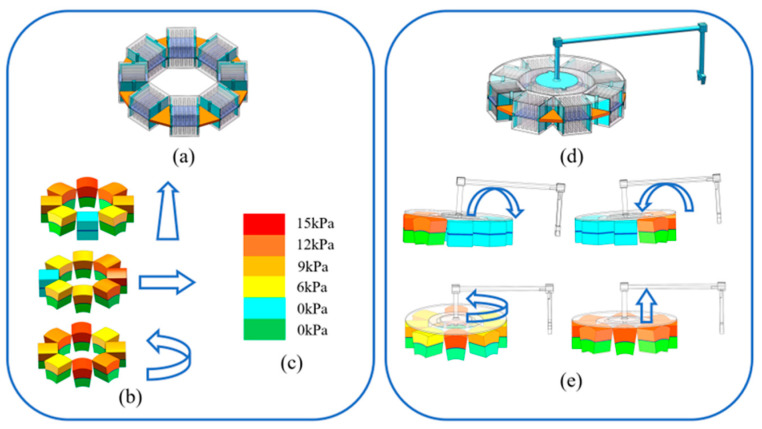
Locomotion and manipulation modes of the MSMP (**a**) 3D model of the MSMP; (**b**) locomotion mode; (**c**) color gradient; (**d**) 3D model of the MSMP with end effector; and (**e**) manipulation mode.

In this paper, the soft actuator units are assembled into a loop-type soft robot that has integrated omnidirectional locomotion and manipulation abilities, as shown in Figure 2a. The side length of the soft actuator unit is 50.0 mm, and the diameter of the loop-type soft robot is 220.7 mm. The omnidirectional locomotion of the robot is realized via the combination of linear locomotion and rotation, as shown in Figure 2b. Both two locomotion modes draw lessons from the transmission principle of a longitudinal wave—just like earthworms and millipedes. Each actuator unit acts as a segment of the whole longitudinal wave, and the wave produces a specific type of rhythm regulated locomotion when the actuator units are allocated phase differences. The phase differences make the soft units show different deformation degrees at a certain moment, and a color gradient is used to illustrate this in Figure 2c. The detailed locomotion principle will be analyzed in Section 3. The MSMP can be equipped with various end effectors, as shown in Figure 2d. The effectors can take the same locomotion modes along with the MSMP to adjust the pose relative to the target objects. Additionally, when the actuator units are inflated, the MSMP can tilt in any direction, changing the orientation of end effectors, just like the manipulation process of parallel robots. The MSMP combines the above motion modes to carry out tasks.

## 3. Locomotion and Deformation Analysis of the MSMP

### 3.1. Locomotion Analysis of the MSMP

As mentioned above, the locomotion principle of the MSMP draws lessons from the transmission principle of a longitudinal wave. To start with, a one-dimensional series structure assembled using the soft actuator units is chosen as the analysis object and example.

**Figure 3:**
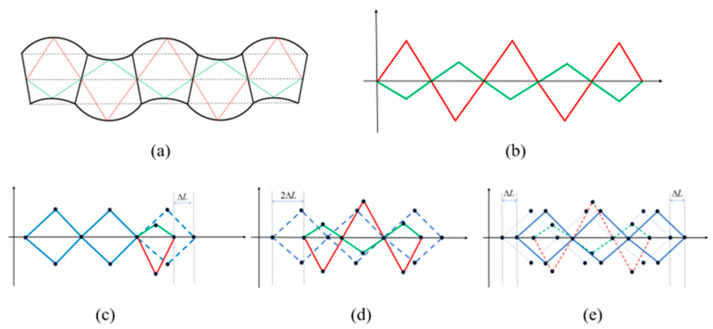
The locomotion principle of the soft modules which is based on the longitudinal wave.

The soft actuator unit is abstracted as a quadrilateral that can be stretched and compressed, with the red border being the inflated half-chamber and the green border being the compressed half-chamber, as shown in Figure 3a,b. The initial state of the soft actuator unit is indicated by the blue border and will be compressed in the direction of the horizontal axis when the upper or lower chamber is inflated, with a compression value is ΔL, as shown in Figure 3c. Additionally, the upper and lower chambers can be inflated simultaneously at certain points during the locomotion process, and the soft actuator unit is indicated by the blue border at this stage since the middle layer of the unit is made of a non-stretchable material.

As shown in Figure 3c,d, the three soft actuator units are actuated sequentially from right to left. The lower half-chamber of the rightmost unit is inflated first, then the upper half-chamber of the middle unit is inflated after a lag, $\varphi_{21}$, with respect to the rightmost unit, and then the lower half-chamber of the leftmost unit is inflated after a lag, $\varphi_{32}$, with respect to the middle unit. Additionally, the inflated half-chambers start to deflate when the deformation reaches the preset amplitude, while the other half-chambers start to inflate. This actuation law has two characteristics:

1. The actuation states are always opposite for two air chambers in one unit.
2. Different half-chambers of two adjacent units have the same actuation state.

**Figure 4:**
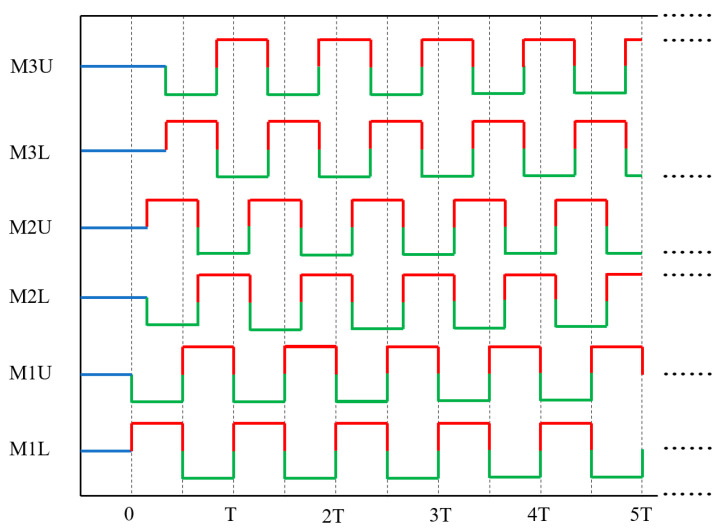
Actuation law of the three-units soft robot.

Figure 4 illustrates the actuation law of the three-unit chain-type soft modular robot. In Figure 4, the red lines indicate the inflating chambers, while the green lines indicate the deflating chambers. M1L and M1U represent the lower and upper chambers of the rightmost unit. T represents the time period required for any half-chamber to complete one expansion and one contraction.

**Figure 5:**
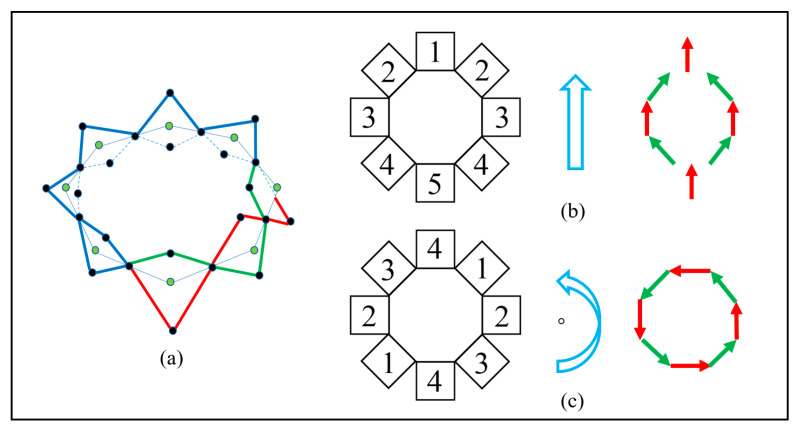
Equivalent locomotion principle of the MSMP. (**a**) the equivalent actuation law in two-dimensional situation; (**b**) the linear locomotion pattern (the numbers indicate the actuation sequence); (**c**) the rotation pattern (the numbers indicate the actuation sequence).

The three-unit system completes a locomotion cycle when the middle layer of the leftmost unit return to the undeformed state and the whole system advances the ΔL to the right, as shown in Figure 3e. The units with a deformed middle layer are equivalent to the dense part of the longitudinal wave, and units with an undeformed middle layer are equivalent to the sparse part. The dense parts make contact with the ground and provide a friction force for the sparse parts that are off the ground to contract; thus, the whole system will locomote forward like a longitudinal wave. The locomotion principle of the chain-type soft modular robot can be generalized to a 2D situation, as shown in Figure 5a–c. The loop-type soft modular robot will perform linear locomotion and rotation according to the same principle.

### 3.2. Deformation Analysis of the Soft Actuator

**Figure 6:**
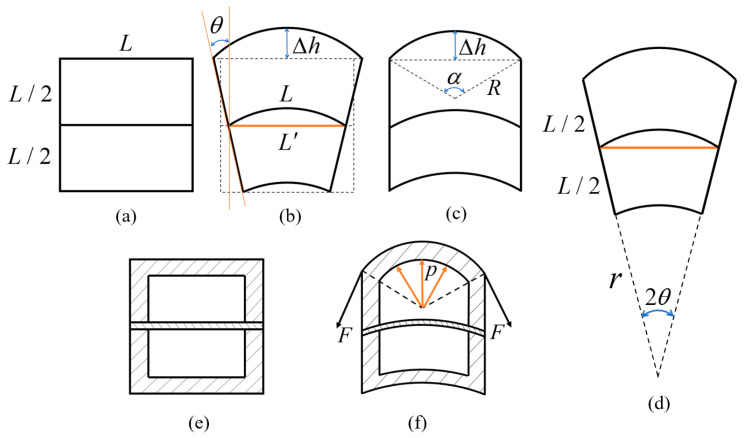
Sketch of the deformation process of the soft actuator unit. (**a**) The main view of the unit when it is not inflated. (**b**,**d**) The main view of the unit when it is inflated. (**c**) The side view of the unit when it is inflated. (**e**,**f**) The sides sectional views of the soft actuator unit.

According to the previous analysis, the deformation of the soft actuator units contributes to the locomotion of the whole system. Therefore, the functional relationship between the deformation of the middle layer and actuation air pressure should be established. As illustrated in Figure 6, the upper half-chamber of the soft unit deforms from the structure shown in Figure 6a to that shown in Figure 6b after inflation. Figure 6e shows the side view of Figure 6a; and Figure 6c,f show the side views of Figure 6b. Additionally, Figure 6e,f are sectional views of the soft actuator unit. To simplify the mathematical model, the following assumptions are put forward:

1. The volume of the silicone material is constant during deformation;
2. In Figure 6b, the three arcs share one circle center, as shown in Figure 6d;
3. The inflation process is in accordance with the ideal gas equation of state.

According to the geometric relations shown in Figure 6a,b,d, the relationship between ΔL and θ can be obtained through Equation (1), and the relationship between Δh and θ can be obtained through Equation (2).

(1) $$\begin{array}{c} \Delta L=L\cdot \left(1-\frac{\sin\theta }{\theta }\right) \end{array}$$

(2) $$\begin{array}{c} \Delta h=\frac{L\cdot \left(1\;+\;\theta \right)\cdot \left(1\;-\;\cos\theta \right)}{2\theta } \end{array}$$

where ΔL is the difference between L and $L^{\prime }$. θ is the tilt angle of the connection plate on both sides of the soft actuator unit. Δh is the arch height of the soft actuator unit. ΔL and Δh are the two critical deformation values for the locomotion and manipulation processes. ΔL determines the locomotion velocity and Δh determines the pitch angle. Additionally, it can be seen that ΔL and Δh can be controlled by the tilt angle θ. Since θ is determined by the inflation air pressure, the relationship between the tilt angle and the inflation air pressure should be analyzed.

Figure 6f illustrates the mechanical relations between the air pressure $p_{1}$ and membrane force F which is described by Equation (3). Additionally, the membrane strain, ε, can be calculated via a geometric relation, so the membrane force, F, can be obtained according to elastic theory, as described by Equations (4)–(6).

(3) $$\begin{array}{c} 2\cdot F\cdot \sin\frac{\alpha }{2}=\int_{-\frac{\alpha }{2}}^{\frac{\alpha }{2}}p_{1}\cdot c\cdot \cos\delta \cdot Rd\delta \end{array}$$

(4) $$\begin{array}{c} \varepsilon =\frac{\alpha }{2\sin\frac{\alpha }{2}}-1 \end{array}$$

(5) $$\begin{array}{c} \alpha =2\arcsin\frac{4\cdot L\cdot ∆h\left(\theta \right)}{L^{2}\;+\;4\cdot {\left(\Delta h\left(\theta \right)\right)}^{2}} \end{array}$$

(6) $$\begin{array}{c} F=E\cdot \varepsilon \cdot c\cdot \left(\frac{L}{2}-k\right) \end{array}$$

**Figure 7:**
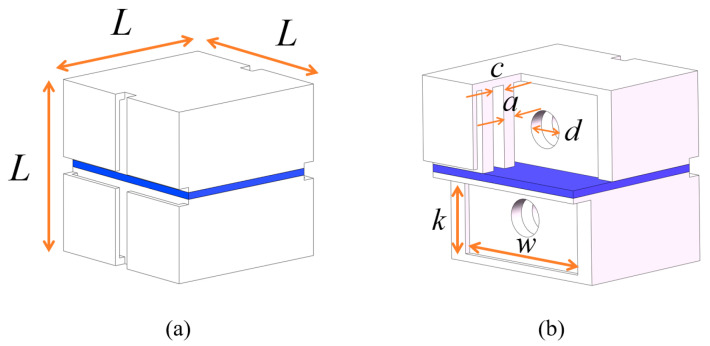
Outline and inner cavity dimensions of the soft actuator unit. (**a**) Outline dimensions of the unit. (**b**) Inner cavity dimensions of the unit.

According to Equations (3)–(6):

(7) $$\begin{array}{c} p_{1}=E\cdot \left(\alpha \left(\Delta h\left(\theta \right)\right)-2\cdot \sin\frac{\alpha \left(\Delta h\left(\theta \right)\right)}{2}\right)\cdot \left(\frac{L}{2w}-\frac{k}{w}\right) \end{array}$$

where $p_{1}$ is the inner air pressure of the soft actuator unit. E is Young’s modulus of the silicone material which is measured using a tensile test. α is the central angle of the top arch in the side view, as shown in Figure 6c. L,k,w are the geometric dimensions of the air cavities inside the half-chamber, as illustrated in Figure 7b.

**Figure 8:**
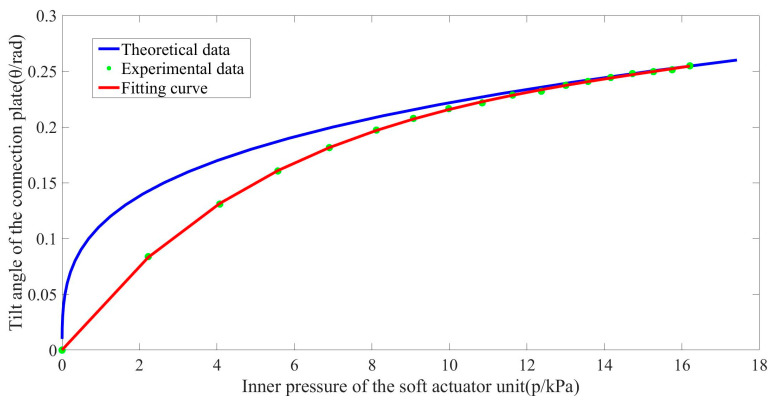
A mathematical model and experiment curve of the relationship between the tilt angle of the connection plate with inner air pressure of the soft actuator unit.

The relation between the tilt angle, θ, of the connection plate and the inner air pressure of the soft actuator unit, $p_{1}$, is described in Figure 8 according to Equations (2), (5) and (7). The data measured through the experiment are also plotted in Figure 8 to verify the accuracy of the mathematical model. The mathematical model fits the experimental curve well when the inner pressure of the soft actuator unit is over 12 kPa. The deviation in the low-pressure area is partly due to the simplification of the elastic modulus. Silicone materials are nonlinear, however in order to keep the model straightforward, it is treated as a constant. Additionally, the three circular arcs formed by the deformation are thought to possess the same circle center when utilizing the geometric model to analyze the deformation characteristics of the soft unit. This assumption will also cause the model to differ from the experiment data. This is due to the possibility that the deformation characteristic of the soft unit may not comply with the law when deformation is small. Nevertheless, the deviation has a slight effect when the model is applied since the soft unit is usually used at 12–16 kPa.

**Figure 9:**
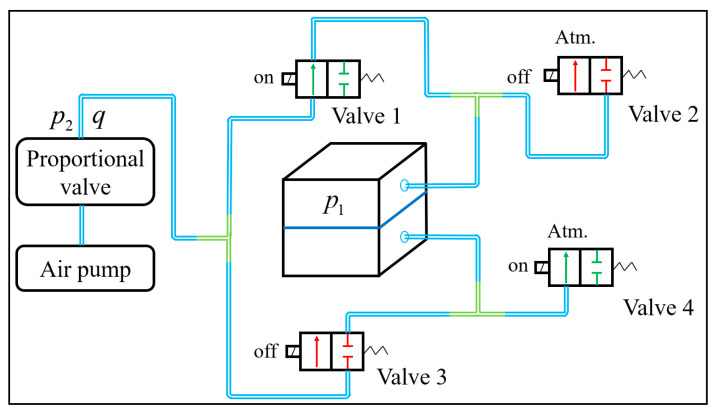
Air pressure control scheme of the soft actuator unit.

Although the mathematical model can describe the relationship between the inner pressure and deformation value of the soft actuator unit, the inner pressure $p_{1}$ cannot be controlled directly. Figure 9 illustrates the setup of the air circuit. The compressed air is transported from the air pump to the proportional valve; regulated by the proportional valve; transported to the solenoid valve; and finally transported into the soft unit through the solenoid valve. Therefore, the inner pressure of the soft actuator unit can be controlled by the proportional valve so that the tilting angle of the soft actuator unit can also be controlled. The relationship between the tilting angle θ and the outlet pressure $p_{2}$ of the proportional valve should be established next.

**Figure 10:**
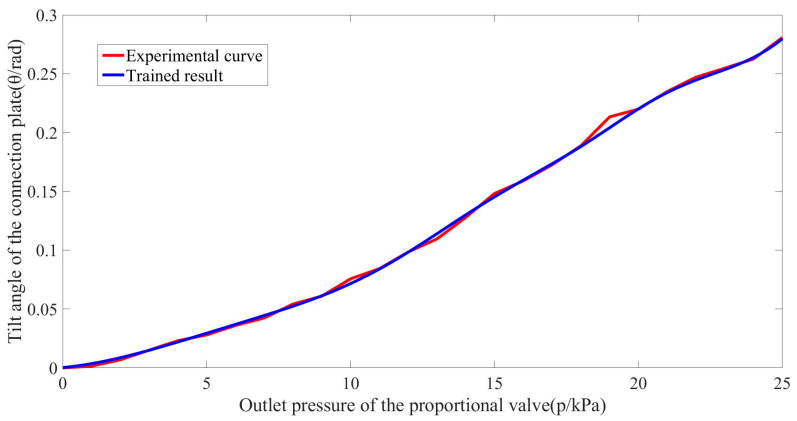
BPNN model and experiment curve of the relationship between the tilt angle of the connection plate with air pressure at the outlet of the proportional valve.

According to the assumption (3):

(8) $$\begin{array}{c} p_{2}=\frac{\left(p_{1}\left(\theta \right)\;+\;p_{a}\right)\cdot V_{1}\left(\theta \right)\;-\;p_{a}\cdot V_{0}}{q\cdot t}-p_{a} \end{array}$$

where $p_{1}$ is the inner air pressure of the soft actuator unit; $p_{a}$ is the atmospheric pressure; $V_{1}$ is the volume of the inner chamber of the soft unit when inflated; $V_{0}$ is the volume of the inner chamber of the soft unit when not inflated; $p_{2}$ is the air pressure at the outlet of the proportional valve; q is the flow rate at the inlet of the solenoid valve; and t is the inflation time, which is a preset value. In Equation (8), the flow rate q is determined by the parameters of the air circuit and the pressure difference between $p_{1}$ and $p_{2}$, making the equation complicated and hard to solve. Therefore, backpropagation neural network (BPNN) is used to establish the relationship between θ and $p_{2}$. The BPNN used in this work has one neuron in the input layer, one neuron in output layer, one hidden layer and five neurons in the hidden layer. The Levenberg–Marquard method [35] is used to train the network. The degree of accuracy of the training is set to be 10^−7^ and the max epochs is set to be 1000. One hundred sets of experimental data are collected and used to train and validate the BPNN. The training result obtained that is between θ and $p_{2}$ is shown in Figure 10 and fits well with the experimental curve. The performance of the BPNN is evaluated by the mean squared error, and the value is 0.048.

## 4. Experimental Results

### 4.1. Experimental Platform Setup

In our experimental system, a PC is used as the upper control computer, and an Arduino Mega2560 is used as the secondary computer for transmitting data and controlling the valves. A monocular vision sensor (KS5A136, Kingcent Inc., Shenzhen, China) is used to provide position information on the soft robot and target objects. Inertial mass units (IMU) (JY61P, Wit Inc., Shenzhen, China) are used to measure and feedback the tilt angle of the soft actuator unit. An air pump (P30TC, Werther Inc., Reggio Emilia, Italy) supplies compressed air to the whole system. Proportional valves (VPPM, Festo Inc., Esslingen, Germany) regulate air pressure according to the feedback values. Solenoid valve sets (T103U-FL, OST Inc., Taizhou, China) control the deformation states of the soft actuator units. 

Each air chamber is controlled by two two-way valves, i.e., each soft actuator unit is controlled by four solenoid valves, as shown in Figure 9. The on/off combination of the solenoid valves enables each air chamber to have three states, inflating, deflating, and holding, and the control logic, shown in Table 1.

### 4.2. Control Strategy of Locomotion and Manipulation

The control system consists of two parts: the vison feedback control part and the pressure control part. 

**Figure 11:**
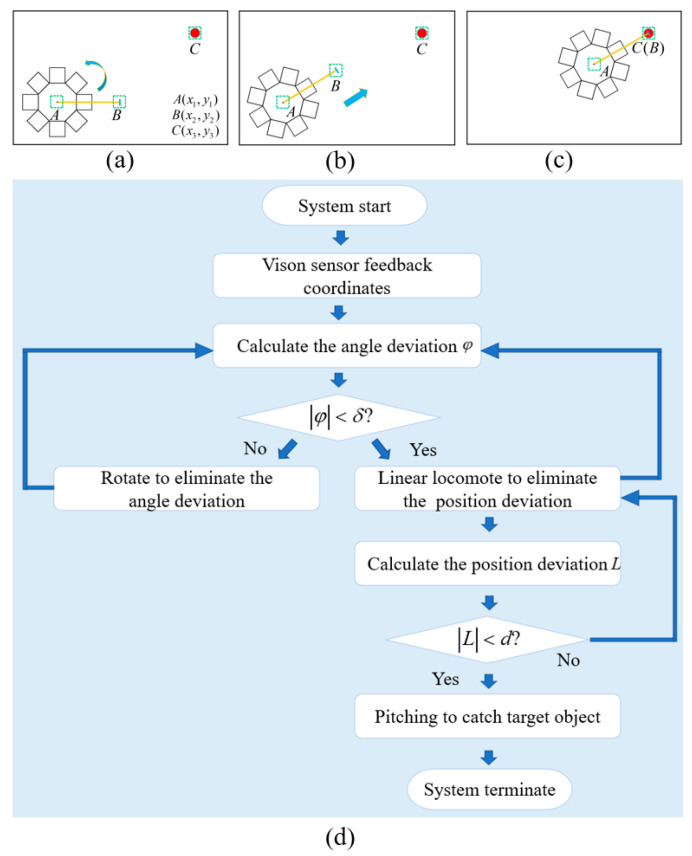
Vision feedback control in the processes of locomotion and manipulation.

In the vision feedback control system, a multi-target tracking algorithm that is integrated into OPENCV is used to track and feedback the positions of the soft robot and target points. The tracking objects are chosen by the SelectROI function provided by OPENCV. The SelectROI function uses bounding boxes to select objects and feedbacks the center coordinates of the boxes regions to the upper computer (PC). Taking Figure 11a–c as an example, point A, point B (on the MSMP), and point C on the target position are chosen as tracking objects. Point A and B construct vector $\overset{⃑}{AB}$ indicates the forward direction of the soft robot. Point A and C construct vector $\overset{⃑}{AC}$ indicates the target direction. The angle between the vector $\overset{⃑}{AB}$ and $\overset{⃑}{AC}$ indicates the angle deviation of the MSMP and the angle value is calculated using the cosine theorem. The position deviation is evaluated via Euclidean distance and is equivalent to the modulus of the $\overset{⃑}{AC}$ vector. Therefore, the position and angle deviations can be calculated using the upper computer according to the coordinates which are used to determine the modes of the MSMP. Subsequently, the upper computer transmits the control command to the sencondary computer (ArduioMega2560). The secondary computer controls the on/off sequences of the solenoid valve set according to the command; thus, controlling the locomotion patterns of the soft robot. Figure 11d shows the flow chart of the control strategy. The MSMP uses linear locomotion to approach the target object and rotates to eliminate the angle deviation. Then, the MSMP switches to manipulation mode and catches the target object.

In the pressure control system, the IMU measures and feedbacks the tilt angle of the soft unit, and then transmits the data to the upper computer. The upper computer calculates the expected air pressure in accordance with the BPNN model. Meanwhile, the proportional valve feedbacks the current air pressure to the upper computer. Then, the upper computer calculates the control pressure according to the expected and current air pressure using the PID control method and transports the control pressure to modify the proportional valve.

### 4.3. Locomotion and Manipulation Experiments

**Figure 12:**
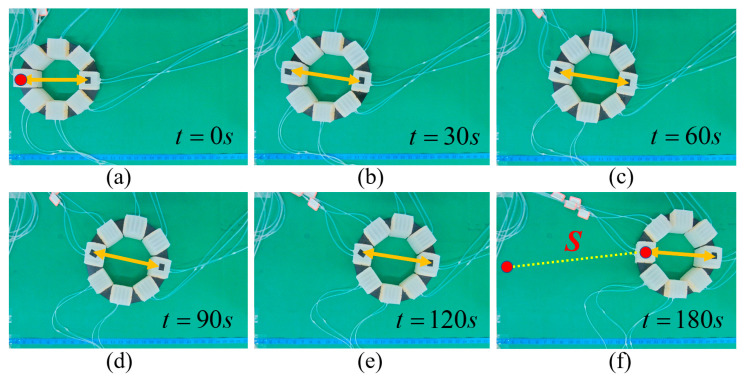
Linear locomotion experiment.

**Figure 13:**
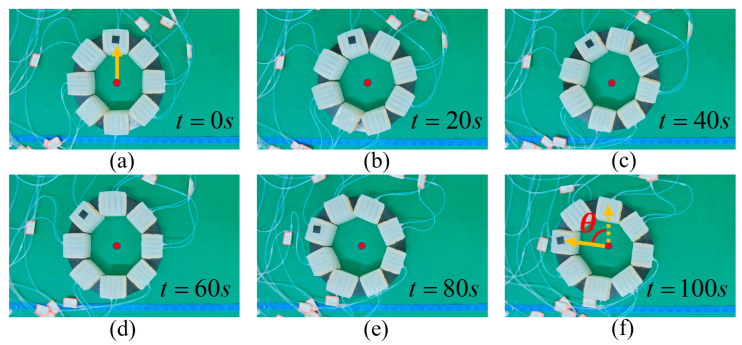
Rotating locomotion experiment.

**Figure 14:**
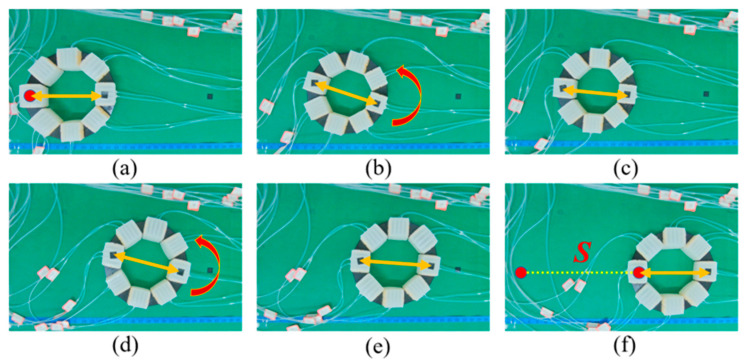
Closed loop locomotion process experiment.

To verify the above theory analysis results, several experiments were conducted. First, linear locomotion and rotation experiments of the MSMP were conducted, as shown in Figure 12 and Figure 13. Figure 12 shows the open loop linear locomotion process. The MSMP moves from left to right taking on a linear locomotion pattern, as illustrated in Figure 5b, and the velocity of linear locomotion is 3.08 mm/s (approximately 0.014 BL/s). It can be observed, from Figure 12, that the orientation of the MSMP changes during the locomotion process. The orientation deviation of the locomotion process is mainly caused by the differences in the soft actuator units during the manufacturing process, which may influence the performance of the soft robot in some special manipulation scenes. Figure 13 shows the rotation process of the MSMP. The rotation pattern is illustrated in Figure 5c and the angular velocity of rotation is 0.71 °/s. It can be observed that manufacturing differences have a slight effect on the rotation process. Therefore, the vision feedback control strategy illustrated in Figure 11 is adopted. The MSMP rotates to eliminate the angle errors that are accumulated by linear locomotion and ensure that it arrives at the target point with the expected orientation. As shown in Figure 14, the black dot on the right indicates the target point; the orange double-headed arrow indicates the orientation of the MSMP; the angle deviations are eliminated during the linear locomotion process; and the orientations of the MSMP are the same in the initial and end stages.

**Figure 15:**
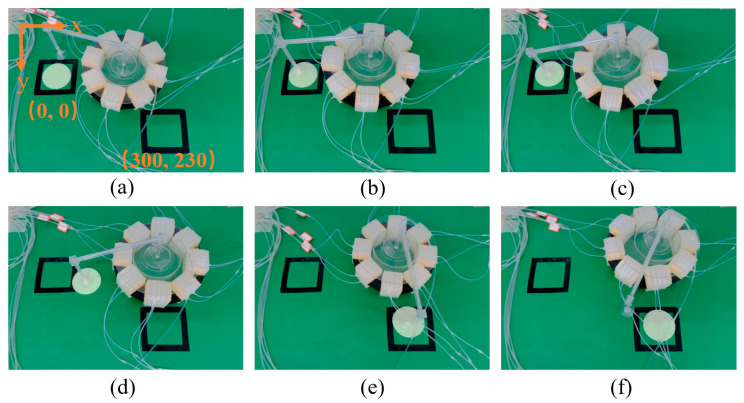
End effector orientation manipulation experiment.

In order to express the manipulation ability of the MSMP, a circular platform with an end sucker is equipped on the MSMP. The sucker is actuated by negative pressure so that it can suck and place objects down. In this experiment, the soft robot needs to transport the circular part from $\left(0,0\right)$ to $\left(300,230\right)$. It is to be noticed that the length of the horizontal arm is 230 mm; thus, the MSMP needs to carry the part and take linear locomotion to arrive at $\left(300,0\right)$ and then rotate in place and carry the part to arrive at $\left(300,230\right)$. As shown in Figure 15a,b, the MSMP rotates to change the pose of the end sucker to approach the target object. Then, the MSMP manipulates the sucker to suck up the object, as shown in Figure 15c. In this process, the MSMP can provide a maximum tilting angle of 7.2°. Figure 15d,e show the process through which the MSMP carries and transports the object from $\left(0,0\right)$ to $\left(300,230\right)$. In Figure 15f, the sucker puts down the object and rotates back along with the MSMP. The circular part is transported from the initial position to the object position with a negligible position error by the assistance of vision sensor.

## 5. Discussion

In this paper, a pneumatic soft actuator unit made of silicone rubber with bi-directional bending ability is proposed. The finite element analysis software ABAQUS is used to observe the deformation characteristics and to optimize the structure of the actuator unit. A mathematical model describing the deformation value of a single unit using inner pressure and a BP neural network describing the deformation value of a single unit using control pressure are established. Experiments are conducted to verify the accuracy of the mathematical and neural network models. In this work, a novel loop-type modular soft robot is put forward which is called mobile soft manipulation platform (MSMP). The MSMP is assembled by several homogeneous actuator units, and the configuration of the robot can be expediently reassembled by changing the relative positions of the actuator units. Compared to the previous soft robot designs, our soft robot has the characteristics of being reconfigurable and reusable. Due to the connection mechanism we designed, the soft actuator units can be extended in three-dimensional directions to constitute more configurations. Also, our soft robot has integrated omnidirectional locomotion and manipulation abilities and this integration feature is seldom focused on in previous works. A locomotion principle based on a longitudinal wave of a multi-unit system is put forward and is applicable not only for series structures but also for 2D structures. Several experiments are carried out to verify the locomotion and manipulation abilities of the MSMP. The experiment results indicate that the MSMP has a linear locomotion velocity of 0.014 BL/s and a rotation angular velocity of 0.71 °/s. The MSMP can provide a maximum tilting angle of 7.2° in the manipulation mode. Compared with previous similar works, the locomotion velocity of the MSMP is not very outstanding since the highlights of our work are its reconfiguration ability, integrated locomotion and manipulation abilities. For example, the soft modular robot proposed by Wang et al. [3] has a linear locomotion velocity of 0.013 BL/s and a rotation angular velocity of 0.71 °/s. The robot put forward by Zou et al. [32] has a linear locomotion velocity of 0.029 BL/s and a rotation angular velocity of 1.63 °/s. And in [34], the soft robot has a linear locomotion velocity of 0.015 BL/s. Also, a vision sensor and several IMU sensors are set up in the experiments to control the locomotion and manipulation processes in a closed loop, which is also an advantage over similar works. Nonetheless, there are still some limitations of the presented work. For instance, the global vision sensor applied in this work is not suitable for practical application in some situations, and the target tracking algorithm used in the control method limits the omnidirectional locomotion ability of the MSMP. Besides, the manipulation ability of the MSMP is limited and needs to be further explored.

In the future, we will focus on improving the locomotion velocity of the soft robot and the integration level of the system, such as by embedding a tracking camera in the soft robot to displace the global vision sensor and exploring the possibility of an untethered system. Additionally, we will further improve the manipulation ability of our soft robot by applying advanced control methods to improve the control accuracy of the end effector and explore more application situations. The soft actuator unit with uniform structure proposed in this paper can be assembled into various configurations, which has extensive application prospects to conduct tasks and is able to adapt to different environments. For example, a soft manipulator and soft gripper have the characteristics of being contact-safe and can be used to operate fragile objects and interact with people safely; soft snakes and worms can be used to explore unknown, narrow, unstructured terrains; and a soft quadruped robot can be used to transport the wounded to rescue sites, with other prospects being possible as well. As a result, we will focus on exploring a general design and control framework for this system.

## Figures and Tables

**Table 1:** Control logic of the solenoid valve.

Valve 1	Valve 2	Valve 3	Valve 4	Upper Chamber	Lower Chamber
on	off	off	on	inflating	delating
off	on	on	off	delating	inflating
off	off	off	off	holding	holding

## Data Availability

Not applicable.
